# Focal Adhesion Kinase Inhibition Contributes to Tumor Cell Survival and Motility in Neuroblastoma Patient-Derived Xenografts

**DOI:** 10.1038/s41598-019-49853-z

**Published:** 2019-09-13

**Authors:** Laura L. Stafman, Adele P. Williams, Raoud Marayati, Jamie M. Aye, Hooper R. Markert, Evan F. Garner, Colin H. Quinn, Shoeb B. Lallani, Jerry E. Stewart, Karina J. Yoon, Kimberly Whelan, Elizabeth A. Beierle

**Affiliations:** 1 Division of Pediatric Surgery, Department of Surgery, University of Alabama, Birmingham, AL 35205 USA; 2 Division of Pediatric Hematology Oncology, Department of Pediatrics, University of Alabama, Birmingham, AL 35233 USA; 3 Department of Cell Developmental and Integrative Biology, University of Alabama at Birmingham, Birmingham, AL 35233 USA; 4 Department of Pharmacology and Toxicology, University of Alabama, Birmingham, AL 35233 USA

**Keywords:** Paediatric cancer, Cancer stem cells, Paediatric cancer

## Abstract

Patient-derived xenografts (PDXs) provide an opportunity to evaluate the effects of therapies in an environment that more closely resembles the human condition than that seen with long-term passage cell lines. In the current studies, we investigated the effects of FAK inhibition on two neuroblastoma PDXs *in vitro*. Cells were treated with two small molecule inhibitors of FAK, PF-573,228 (PF) and 1,2,4,5-benzentetraamine tetrahydrochloride (Y15). Following FAK inhibition, cell survival and proliferation decreased significantly and cell cycle arrest was seen in both cell lines. Migration and invasion assays were used to determine the effect of FAK inhibition on cell motility, which decreased significantly in both cell lines in the presence of either inhibitor. Finally, tumor cell stemness following FAK inhibition was evaluated with extreme limiting dilution assays as well as with immunoblotting and quantitative real-time PCR for the expression of stem cell markers. FAK inhibition decreased formation of tumorspheres and resulted in a corresponding decrease in established stem cell markers. FAK inhibition decreased many characteristics of the malignant phenotype, including cancer stem cell like features in neuroblastoma PDXs, making FAK a candidate for further investigation as a potential target for neuroblastoma therapy.

## Introduction

Neuroblastoma, the most common pediatric extracranial solid tumor, is derived from neural crest cells, and patients with non-high-risk disease benefit greatly from the current therapeutic options available. Unfortunately, five-year survival remains less than 50% for patients with high-risk disease^1^, and current treatment regimens are associated with substantial morbidity. Further research is needed to identify new pathways and targets that may lead to novel therapies. The investigation of these novel therapies depends on reliable experimental models that recapitulate those properties of the tumors seen in patients.

Focal adhesion kinase (FAK), a non-receptor tyrosine kinase, has become a target of interest in several malignancies including colon, breast, and lung carcinomas^2^, as well as neuroblastoma^3^ due to its role in cell survival, proliferation, and motility^3^. FAK has been shown to activate pro-survival pathways such as Ras-ERK and PI3K-AKT, while having an anti-apoptotic effect through the degradation of p53^4^. FAK acts as an important cell signaling scaffold that when phosphorylated, activates downstream pathways such as SRC family kinases and ERK, both of which affect cell cycle and cell motility^5^. In neuroblastoma, FAK overexpression has been linked to a more aggressive phenotype^6^. We previously demonstrated that inhibition of FAK decreased neuroblastoma cell survival, migration, invasion, and metastases using long-term passage neuroblastoma cell lines with *MYCN* amplification^7^. *MYCN*, which is the most important negative prognostic factor in neuroblastoma, has been demonstrated to bind the FAK promoter, subsequently increasing the production of FAK in *MYCN* amplified cells; thus, enhancing its role in this particular malignancy^3^. For this reason, we sought to characterize the role of FAK in *MYCN* amplified patient-derived xenografts (PDXs).

The PDXs investigated in this study, COA3 and COA6, were *MYCN* amplified tumors, derived from primary tumors in children with high risk disease. Neuroblastomas with both MYCN and FAK have been found to be over-expressed in patients with higher risk disease and poorer prognosis^6^. Additional mutations were identified with full exome sequencing but were chosen not to be investigated because of the clinical significance of *MYCN* amplification^8^. Thus, COA3 and COA6 were optimal PDXs to evaluate the effects of FAK inhibition; however, both of these tumors took two to six months to propagate in animals thereby making them practical only for *in vitro* experimentation.

The majority of data surrounding the role of FAK in cancer has been generated from studies utilizing long-term passage cell lines that have been selected to escape senescence. These readily available cells are critical tools for amassing data in the preclinical phase that create a foundation for further investigation; however, when available, PDXs often more closely recapitulate the actual human condition^9^. The novelty of the present study is the use of unique human neuroblastoma PDXs that are *MYCN* amplified to evaluate the effects of FAK inhibition on neuroblastoma cells *in vitro*.

## Results

### FAK Inhibition Decreased PDX Survival and Proliferation *in vitro*

**Figure 1:**
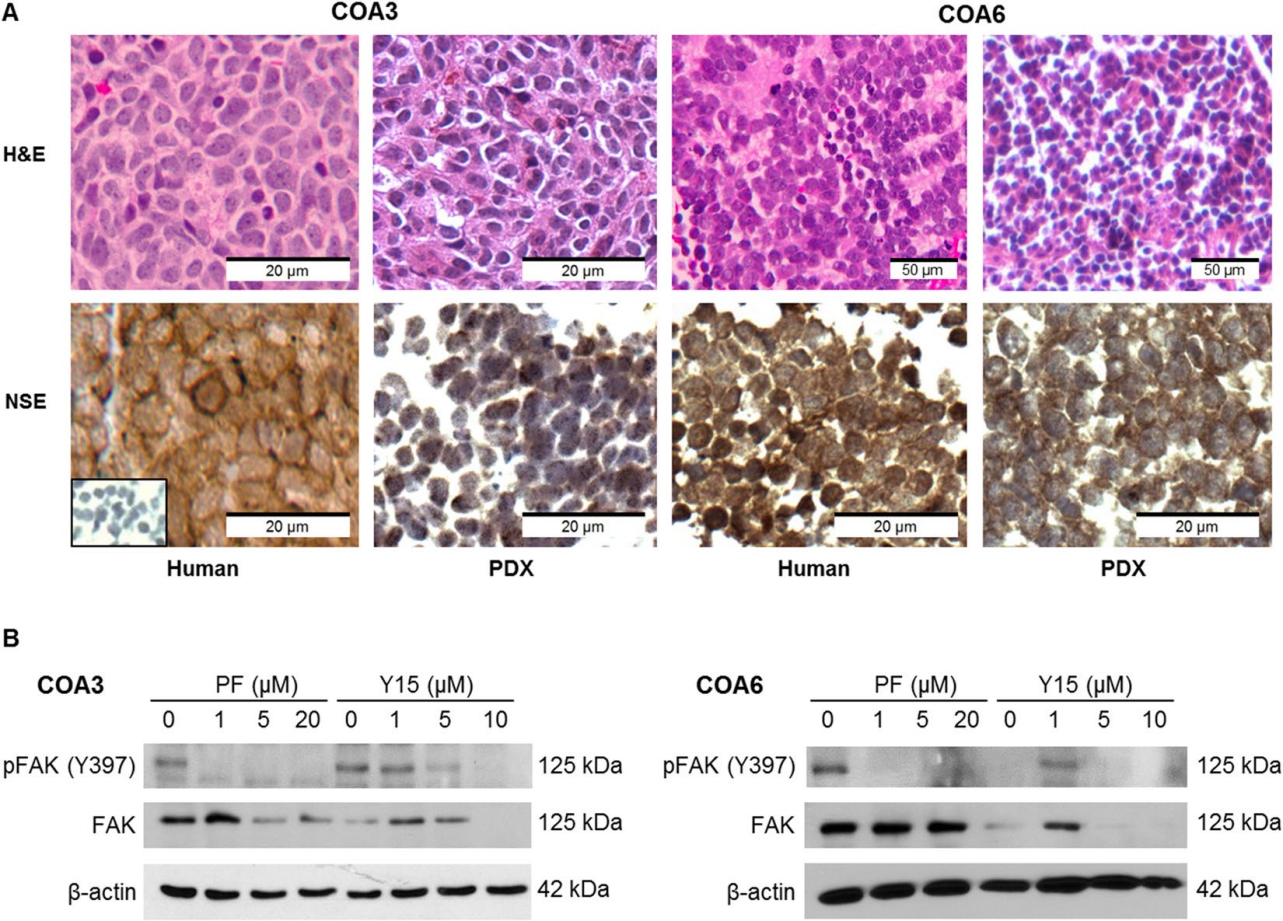
Human patient derived xenografts recapitulate the human tumor of origin and express FAK. (**A**) Hematoxylin and eosin staining (*top panels*) of the human specimens (*left*) and the tumors harvested from the mice (*right*) showed that the PDX tumors recapitulated the histology of the human specimens from which they were derived. Immunohistochemistry staining for neuron-specific enolase (*bottom panels*), a marker for neuroendocrine neoplasms, showed similar staining between the human specimens and the PDX tumors. (**B**) Immunoblotting of tumor lysates for total and phosphorylated (Y397) FAK demonstrated baseline expression of FAK and pFAK (Y397) in both COA3 and COA6 cells. In both cell lines treatment with FAK inhibitors PF and Y15 resulted in a decrease in both phosphorylated and total FAK in a dose-dependent manner.

Two human neuroblastoma patient-derived xenografts, COA3 and COA6, were utilized for experimentation. Hematoxylin and eosin (H&E) staining verified that the PDXs recapitulate the primary human tumors from which they were derived (Fig. 1A, *upper panels*). Further, staining with neuron-specific enolase (NSE), a marker for neuroblastoma, demonstrated positive staining in both the primary tumors and the PDXs (Fig. 1A, *lower panels*) further confirming the PDXs replicated the parent human tumor.

Cells were treated with two small molecule FAK inhibitors, PF-573,228 (PF) and 1,2,4,5-benzentetraamine tetrahydrochloride (Y15). Immunoblotting confirmed decreased expression of FAK protein following treatment with either inhibitor in both PDXs (Fig. 1B). Phosphorylation of FAK at the Y397 site followed the same trend as total FAK.

**Figure 2:**
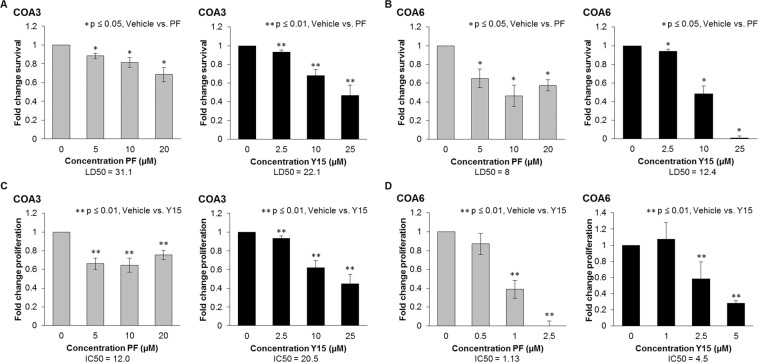
FAK inhibition with PF and Y15 decreased COA3 and COA6 cell survival and proliferation. (**A**) COA3 cells (1.5 × 10^4^) were treated with increasing concentrations of PF (0, 5, 10, 20 μM) or Y15 (0, 2.5, 10, 25 μM) for 24 hours and cell survival was measured using alamarBlue® assay. There was a significant decrease in cell survival following treatment with PF and Y15, with LD_50_ of 31.1 and 22.1 μM respectively. (**B**) COA6 cells (1.5 × 10^4^) were treated in the same fashion, and there was a significant decrease in cell survival following treatment with PF and Y15, with LD_50_ of 8.0 and 12.4 μM respectively. (**C**) COA3 cells (5 × 10^3^) were treated with increasing concentrations of PF (0, 5, 10, 20 μM) or Y15 (0, 2.5, 10, 25 μM) for 24 hours and cell proliferation was measured using CellTiter96 assay®. Proliferation was significantly decreased following treatment with PF and Y15, with IC_50_ of 12.0 and 20.5 μM respectively. (**D**) COA6 cells (5 × 10^3^) were treated with lower doses of PF (0, 0.5, 1, 2.5 μM) and Y15 (0, 1, 2.5, 5 μM) due to increased sensitivity. Proliferation was significantly decreased following treatment with PF and Y15, with IC_50_ of 1.13 and 4.5 μM, respectively. Data represent at least three biologic replicates and are reported as mean ± SEM.

FAK inhibition has been shown to decrease cell viability and proliferation in long-term passage neuroblastoma cell lines^7^. To determine if human neuroblastoma PDX cells were affected by small molecule FAK inhibition, alamarBlue® assay was utilized to measure the effects of PF and Y15 on viability in the COA3 and COA6 PDX cells. FAK inhibition resulted in a significant decrease in survival for both COA3 (Fig. 2A) and COA6 (Fig. 2B) cells. Lethal dose 50% (LD_50_) was variable between agents and cell lines (Fig. 2A,B). Similar findings were seen with proliferation as evaluated by CellTiter96® assay. Proliferation was significantly diminished in both neuroblastoma PDXs following inhibition of FAK and inhibitory concentration 50% (IC_50_) varied between agents and cell lines (Fig. 2C,D).

### FAK inhibition inhibited progression through the cell cycle

**Figure 3:**
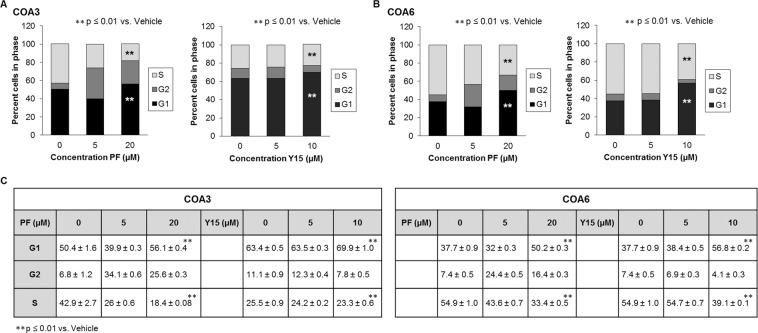
FAK inhibition diminished progression through the cell cycle. (**A**) COA3 cells (3 × 10^6^) were treated with increasing doses of PF (0, 5, 20 μM) or Y15 (0, 5, 10 μM) for 72 hours. Cells were stained with propidium iodide and cell cycle was analyzed via flow cytometry. FAK inhibition led to an increase in percentage of cells in the G1 phase and a decrease in percentage of cells in S phase. This change was statistically significant and represents failure to progress through the cell cycle. (**B**) COA6 cells (3 × 10^6^) were treated with increasing doses of PF (0, 5, 20 μM) or Y15 (0, 5, 10 μM) for 72 hours. Cells were stained with propidium iodide and cell cycle was analyzed via flow cytometry. FAK inhibition led to an increase in percentage of cells in the G1 phase and a decrease in percentage of cells in S phase. This change was also statistically significant, representing failure to progress through the cell cycle. (**C**) Cell cycle data are presented in tabular form. Data represent at least three biologic replicates and are reported as mean ± SEM.

To further investigate the mechanism by which FAK inhibitors decreased neuroblastoma cell survival and proliferation, we examined the cell cycle. Previous studies in human fibroblasts have demonstrated that overexpression of FAK resulted in acceleration of cell progression from the G1 to S phase, while knockout of FAK resulted in decreased progression from the G1 to S phase, so we anticipated similar results in the PDXs^10^. There was a significant increase in percentage of cells in G1 phase with an accompanying decrease in percentage of cells in S phase after treatment with PF or Y15 in both the COA3 (Fig. 3A) and COA6 (Fig. 3B) PDX cells. Graphic data are presented in tabular form in Fig. 3C. Representative histograms are presented in Supplementary Data Fig. 1. These results indicated that FAK inhibition in the PDXs resulted in a failure of the cells to progress through the cell cycle, stalling in the G1 phase.

### FAK inhibition decreased invasion and migration

**Figure 4:**
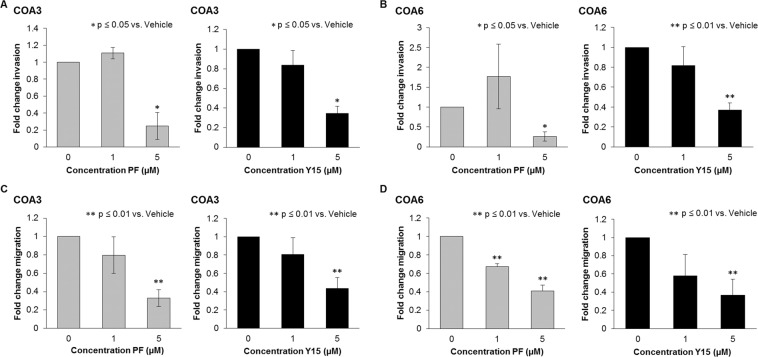
FAK inhibition decreased invasion and migration of COA3 and COA6 PDX cells. (**A**) COA3 cells (3 × 10^6^) were treated for 24 hours with increasing concentrations of PF (0, 1, 5 μM) and Y15 (0, 1, 5 μM) and plated in Transwell plates. Cells were allowed to invade for one week through a Matrigel layer. Treatment with both PF and Y15 resulted in a statistically significant decrease in COA3 cell invasion. (**B**) COA6 cells (4 × 10^4^) were treated for 24 hours with increasing concentrations of PF and Y15 and plated in Transwell plates. Cells were allowed to invade for one week through a Matrigel layer. Treatment with both PF and Y15 resulted in a statistically significant decrease in COA6 cell invasion. (**C**) COA3 cells (3 × 10^6^) were treated with increasing concentrations of PF (0, 1, 5 μM) and Y15 (0, 1, 5 μM) for 24 hours, plated into Transwell migration plates, and allowed to migrate for 72 hours. Treatment with both PF and Y15 resulted in a statistically significant decrease in COA3 cell migration. (**D**) COA6 cells (4 × 10^4^) were treated for 24 hours, plated into Transwell migration plates and allowed to migrate for 72 hours. Treatment with both PF and Y15 resulted in a statistically significant decrease in COA6 cell migration. Data represent at least three biologic replicates and are reported as mean ± SEM.

Inhibition of FAK has been noted to decrease the motility of neuroblastoma cells^11^. To determine whether neuroblastoma PDX cells were similarly affected, we utilized modified Boyden chamber assays for invasion and migration. For both PDXs, PF and Y15 significantly decreased the ability of the cells to invade (Fig. 4A,B). Similar results were noted with migration (Fig. 4C,D). These findings suggested that FAK affected motility in neuroblastoma PDX cells.

### FAK inhibition decreased tumor cell stemness

**Figure 5:**
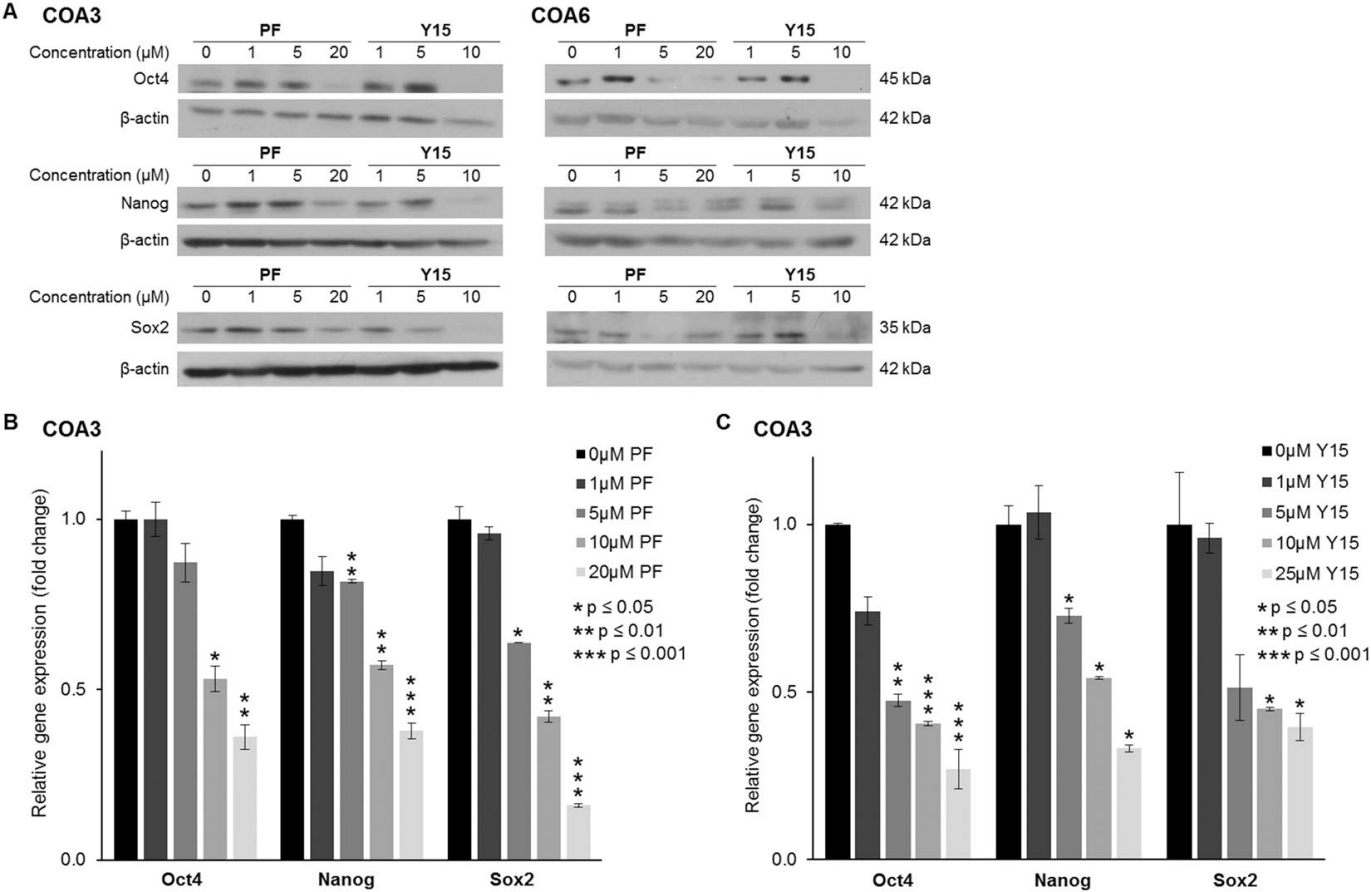
FAK inhibition decreased tumor cell stemness. (**A**) Immunoblotting of COA3 and COA6 cell lysates for markers of stemness including Oct4, Sox2, and nanog was performed. Treatment of cells with increasing concentrations of PF or Y15 resulted in decreased expression of these markers in both the COA3 and COA6 cells. (**B**) Quantitative real-time PCR analysis of COA3 cells was performed to evaluate mRNA expression of Oct4, nanog, and Sox2. Treatment of COA3 cells with increasing concentrations of PF or Y15 resulted in decreased fold change expression of these three markers.

**Figure 6:**
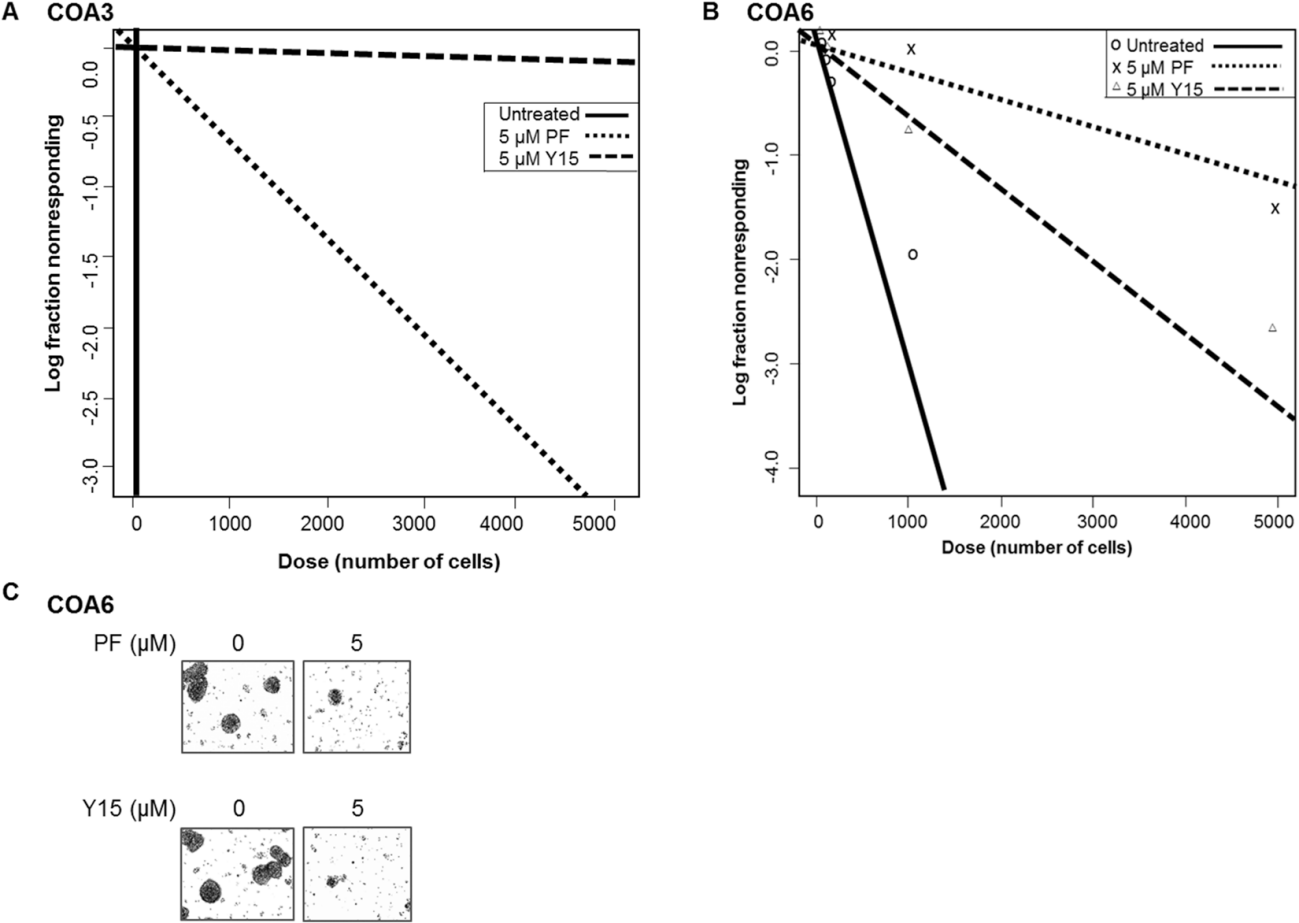
FAK inhibition decreased tumorsphere forming capacity. Single cells of (**A**). COA3 or (**B**). COA6 were plated in conditioned NB media onto 96-well low attachment plates using serial dilutions with 5000, 1000, 100, 50, 20, 10, and 1 cell per well. Cells were then treated with PF (0, 5 μM) or Y15 (0, 5 μM). After one week the number of wells containing tumor spheres in each row was counted by a single researcher and the results were analyzed using the extreme limiting dilution analysis software (http://bioinf.wehi.edu.au/software/elda/). After one week, photos of the wells were obtained. Treatment with 5 μM of either PF or Y15 caused a significant decrease in the tumorsphere formation in COA3 (**A**) and COA6 cells (**B**), indicating a decrease in SCLCC phenotype. **C**. Representative photographs demonstrate decreased number of tumorspheres following FAK inhibition with PF (top panel) or Y15 (bottom panel).

FAK has been noted to support the cancer stem cell phenotype in other cancer cell lines^4^. We wished to determine if FAK supported a stem cell-like cancer cell (SCLCC) phenotype in the neuroblastoma PDX cells. First, specific stem cell markers associated with embryonic pleuripotency were investigated^12^. Sox2, Oct4, and nanog are three transcription factors that are markers for dedifferentiated pleuripotent cells, which describes the cell population that we sought to target^12^. Immunoblotting showed a decrease in expression of all three proteins in both COA3 cells and COA6 cells following treatment with either PF or Y15 (Fig. 5A), signifying that FAK inhibition led to decreased expression of stem cell markers. In addition, we used real-time PCR (qPCR) to examine mRNA abundance of the three transcription factors in COA3 cells, which further confirmed decreased mRNA expression of the stem cell markers following treatment with either PF or Y15 (Fig. 5B). Finally, an extreme limiting dilution assay was used to investigate the effect of FAK inhibitors on the SCLCC phenotype. Treatment with either PF or Y15 decreased sphere forming ability of COA3 (Fig. 6A) and COA6 cells (Fig. 6B). Representative photomicrographs of the COA6 spheres are presented in Fig. 6C. These findings provided evidence that FAK inhibition decreased the SCLCC phenotype in these human neuroblastoma PDX cells.

### FAK inhibition decreased survival and proliferation of both CD133-enriched and CD133-depleted cells

**Figure 7:**
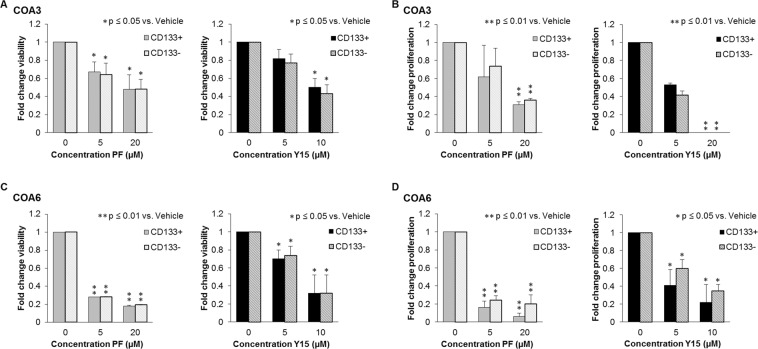
FAK inhibition led to decreased survival and proliferation in COA3 and COA6 sorted cell populations. COA3 and COA6 cells were sorted for CD133 using a magnetic cell sorting system. (**A**) COA3 CD133-enriched or CD133-depleted cells (1.5 × 10^4^) were treated with increasing concentrations of PF (0, 5, 20 μM) or Y15 (0, 5, 10 μM) for 5 days and cell survival was measured using alamarBlue® assay. There was a significant decrease in cell survival in both populations following treatment with PF and Y15. (**B**) COA6 CD133-enriched or CD133-depleted cells (1.5 × 10^4^) were treated in the same fashion, and there was a significant decrease in cell survival following treatment with PF and Y15 in both populations. (**C**) COA3 CD133-enriched or CD133-depleted cells (5 × 10^3^) were treated with increasing concentrations of PF or Y15 for 5 days and cell proliferation was measured using CellTiter96® assay. Proliferation was significantly decreased following treatment with PF and Y15 in both cell populations. (**D**) COA6 CD133-enriched or CD133-depleted cells (5 × 10^3^) were treated in the same fashion, and proliferation was significantly decreased following treatment with PF and Y15 in both cell populations. Data represent at least three biologic replicates and are reported as mean ± SEM.

To study SCLCCs, those cells thought to be responsible for treatment resistance and tumor recurrence, required this cell population to be isolated from the bulk tumor cell population. CD133 has been identified as a marker for tumor cell stemness in neuroblastoma^13^ and served as the marker for the SCLCC cell population for the current study. Magnetic cell sorting for CD133 was performed, resulting in CD133-enriched and CD133-delpleted populations. Subsequently, survival and proliferation assays were performed on the sorted COA3 and COA6 cells after treatment with PF and Y15. In the COA3 cells, PF treatment resulted in a significant decrease in the survival of both the CD133-enriched and CD133-depleted populations at 5 μM. A significant decrease in survival was seen with Y15 treatment at 10 μM (Fig. 7A). Similar results were seen with proliferation in the COA3 cells; a significant decrease following treatment with PF or Y15 (Fig. 7B). Viability and proliferation were both decreased in the COA6 cells following treatment with either FAK inhibitor (Fig. 7C,D). These data demonstrated that the CD133-enriched population was not resistant to FAK inhibition and that the FAK inhibitors effectively targeted the SCLCC population.

### FAK inhibition affected cell cycle progression of both CD133-enriched and CD133-depleted cell populations

**Figure 8:**
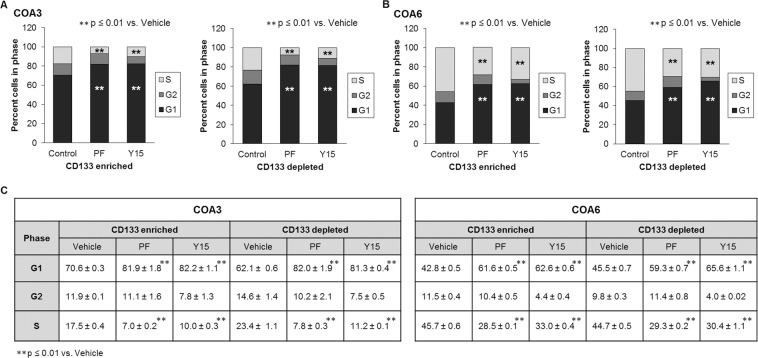
FAK inhibition diminished progression of both CD133-enriched and depleted cell populations through the cell cycle. COA3 and COA6 cells were sorted using a magnetic cell sorting system into CD133-enriched and CD133-depleted population. Cells (3 × 10^6^) were treated with PF (0, 5 μM) or Y15 (0, 5 μM) for 72 hours, stained with propidium iodide and cell cycle was analyzed via flow cytometry. (**A**) FAK inhibition led to an increase in percent of COA3 cells in the G1 phase and a decrease of those in S phase in both the CD133-enriched and depleted populations, representing failure to progress through the cell cycle. (**B**) FAK inhibition led to an increase in percent of COA6 cells in the G1 phase and a decrease of those in S phase in both the CD133-enriched and depleted populations, representing failure to progress through the cell cycle. (**C**) Cell cycle data are presented in tabular form. Data represent at least three biologic replicates and are reported as mean ± SEM.

In order to demonstrate whether CD133-enriched cells, or SCLCC, were resistant to the FAK inhibitors’ effects on cell cycle, analysis was performed on the CD133-enriched and CD133-depleted cell populations. Following magnetic cell sorting, cells treated with PF and Y15 underwent cell cycle analysis. As was seen in the bulk cell population, both the CD133-enriched and CD133-depleted populations showed evidence of failure to progress through the cell cycle. This effect was seen in both populations (Fig. 8A,B). The graphic data are presented in tabular form in Fig. 8C.

## Discussion

We have demonstrated that the inhibition of FAK in human neuroblastoma PDXs decreased the malignant phenotype of the PDX cells *in vitro*. We have previously demonstrated similar results when utilizing long-term passage neuroblastoma cell lines^8^; however, the use of PDXs makes this study novel. PDXs that have been demonstrated to recapitulate the properties of the parent tumor provide a unique instrument for study of an actual tumor *in vitro*. Braekeveldt and Bexell discussed that exposure of long-term passage cell lines to fetal calf serum leads to differentiation and genetic changes that distance cells from the original patient tumor characteristics^14^. Realistically, every tumor carries a unique genetic fingerprint and frequent mutations may make finding one universally applicable therapy challenging. We utilized xenografts from two different patients with known high-risk neuroblastoma in order to validate the role of FAK in this cancer. These data showing that more than a single PDX responded similarly to more than a single FAK inhibitor provided additional evidence to generalize these findings to the disease.

While PDXs are beneficial in the study of novel cancer therapeutics, there are limitations to their use which must be recognized. Braekeveldt and Bexell pointed out three important limitations of the PDX model^14^. First, the necessary use of immunocompromised mice eliminates the thorough study of immunotherapies or immune responses to therapy. Second, the tumor microenvironment in an immunocompromised mouse is markedly different from that of an immunocompetent animal. Third, the heterogeneity of a patient tumor may result in the equivalent of a sampling error when selecting a piece of tumor to inject into a mouse, resulting in a less aggressive or more indolent collection of cells being passaged^14^. Our lab as well as other investigators have previously proposed an additional caution to the PDX model, which is that they require frequent surveillance due to the potential for the development of other tumor types that may not appear grossly different from the intended tumor^15^. In terms of *in vivo* experimentation, these PDXs vary in utility from cell line to cell line. The COA3 and COA6 tumors required between two and six months to grow and did not grow in a consistent or uniform rate, making them inconvenient for *in vivo* experimentation. In spite of these limitations, PDX models provide an approach that considers the intricacies of patient disease and thereby play an important role in pre-clinical study of therapeutics.

The FAK inhibitors chosen for this study, PF and Y15, are designed to prevent auto-phosphorylation of FAK at Y397^7^. In the current study, these compounds were also noted to affect total FAK expression. These findings have been noted by other authors in other cancer cell lines^16,17^. Whether through inhibition of auto-phosphorylation or down-regulation of total FAK, the end result as demonstrated by immunoblotting was less phosphorylated FAK. Through treatment with PF and Y15, effective targeting of active FAK resulted in significant changes in the neuroblastoma PDX cells.

The off-target effects of small molecule inhibitors are well documented^18^ and cannot be ignored as a factor in the current investigations. We demonstrated significant response in the cells lines at doses <10 μM as is recommended to minimize off-target effects, and saw a dose dependent increase in efficacy^18^. Ideal inhibition of FAK protein utilizing other methods such as RNAi or CRISPR knockout were not possible. These PDX cells could not be sustained in the tissue culture environment, making these methods that may have fewer off-target effects impossible to explore.

It is important to highlight that both COA3 and COA6 were *MYCN* amplified tumors. Previous studies have demonstrated the importance of the relation between MYCN and FAK and are indicative of poor prognosis as well as high risk disease^6^. MYCN was demonstrated to bind to the FAK promoter and function to modulate the transcription of FAK^3^. FAK has not been shown to affect the expression or function of *MYCN*. For these reasons, it was critical that the effects seen with FAK inhibitors on MYCN-amplified long-term passage neuroblastoma cells hold true in PDXs, as was demonstrated in the current study.

Cancer cell stemness is an important area of investigation as these cell populations are believed to be responsible for cancer cell resistance and renewal^19,20^. FAK has been shown to play a role in maintaining this cancer stem cell population in other types of malignancy^4,21^, prompting our investigation of its role in maintaining SCLCCs in neuroblastoma. Schober and colleagues showed that integrin binding triggers self-renewal signals through FAK in skin cancer in order to maintain the cancer stem cell population^21^. In a breast cancer study, Luo demonstrated that inhibition of FAK suppressed the self-renewal of breast cancer stem cells^22^. Based on these findings, we hypothesized that FAK inhibition would target the neuroblastoma SCLCC population and reduce stemness. We demonstrated diminished stemness of these neuroblastoma PDX cells following FAK inhibition through decreased tumorsphere formation and expression of stem markers. We were also able to show the efficacy of FAK inhibition on survival, proliferation, and progression to mitosis in the SCLCC population. In order for therapeutic interventions to be more effective, it is imperative that the SCLCC population does not escape the effects of treatment. The data presented here demonstrated that the CD133-enriched population (SCLCC) was as sensitive to the effects of both FAK small molecule inhibitors as the CD133-depleted (non-SCLSS) population.

**Figure 9:**
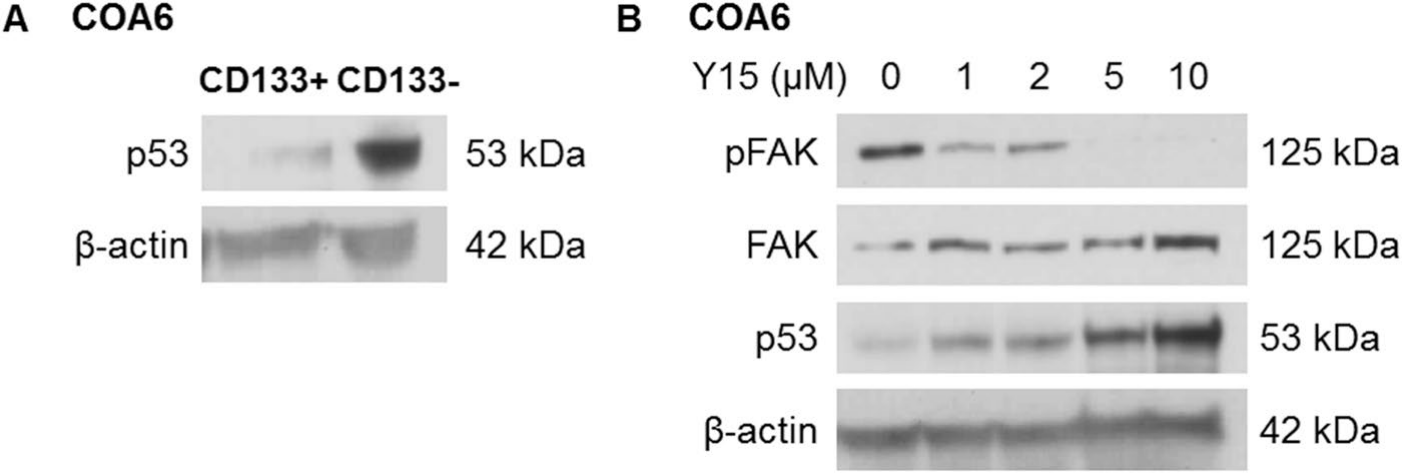
p53 expression in neuroblastoma PDX SCLCC. (**A**) Immunoblotting for p53 of CD133 sorted COA6 cell lysates revealed decreased expression of p53 in the CD133-enriched (CD133+) compared to the CD133-depleted (CD133−) cell population. (**B**) COA6 unsorted tumor cells were treated with increasing concentrations of PF for 24 hours. Whole cell lysates were subjected gel electrophoresis and immunoblotting completed for p53. Expression of p53 increased as pFAK expression decreased. Expression of β-actin confirmed equal protein loading.

We propose that the p53 pathway may be one mechanism by which FAK inhibition targets neuroblastoma SCLCC. Although p53 is rarely mutated in neuroblastoma, it is usually not functional. Oh and colleagues suggested that the dysfunction of p53 may be secondary to downregulation or inactivation through other mechanisms, such as the inhibition of upstream transcription factors, epigenetic modifiers, or post-translational changes^23^. In general, human stem cells maintain low expression of p53 as a result of negative regulators such as MDM2, in order to maintain their potential for self-renewal and escape apoptosis^24^. We found a lower baseline expression of p53 in the CD133-enriched SCLCCs compared to CD133-depleted population (Fig. 9A). We previously demonstrated that inhibition of FAK in long term passage neuroblastoma cell lines led to an increase in p53 expression, suggesting that FAK plays a role in downregulating p53^25^. When COA6 PDX cells were treated with FAK inhibitors, we noted an increase in p53 expression at increasing doses of Y15 (Fig. 9B). We hypothesize that this increase in p53 expression may be a mechanism by which FAK inhibition effectively targets the SCLCC population.

Using human neuroblastoma PDX models, this study validates previous studies in long-term passage cell lines that demonstrated the efficacy of FAK inhibition in neuroblastoma. Patients who have the greatest need for novel therapeutic options are those with high-risk disease. By evaluating this therapy on xenografts derived from patients with high-risk disease, we are modeling our target treatment group as closely as possible *in vitro*. Moving forward, FAK inhibition warrants further evaluation for neuroblastoma therapy.

## Methods

### Cell culture

Two human neuroblastoma PDXs, COA3 and COA6, were utilized for experimentation. These PDXs were developed as described below. Individual cells were obtained by dissociating the COA3 and COA6 xenograft tumors using a Tumor Dissociation Kit (Miltenyi Biotec, San Diego, CA) per manufacturer’s protocol, and resuspended in neurobasal medium (NB) (Life Technologies, Carlsbad, CA) supplemented with B-27 without Vitamin A (Life Technologies), N2 (Life Technologies), amphotericin B (250  μg/mL), gentamicin (50μg/mL), L-glutamine (2 mM), epidermal growth factor (10 ng/mL; Miltenyi Biotec), and fibroblast growth factor (10 ng/mL; Miltenyi Biotec). Following dissociation, cells were maintained at 37 °C with 5% CO_2_ overnight prior to use for experimentation. Both COA3 and COA6 cells were verified within the last 12 months using short tandem repeat analysis [Heflin Center for Genomic Sciences, University of Alabama, Birmingham, (UAB), Birmingham, AL]. Real-time qPCR was performed to assess the percentage of human and mouse DNA contained in the COA3 and COA6 PDXs to ensure that the tumors did not harbor murine contamination (TRENDD RNA/DNA Isolation and TaqMan QPCR/Genotyping Core Facility, UAB, Birmingham, AL).

### Establishing patient-derived xenografts

The study was approved by the UAB Institutional Review Board (X130627006) and UAB Institutional Animal Care and Use Committee (IACUC-09803). After obtaining written informed consent, fresh tissue was obtained from pediatric patients with primary neuroblastoma tumors undergoing surgical excision. An additional piece of the surgical specimen was placed in formalin and embedded in paraffin for immunohistochemistry. To establish the COA3 and COA6 PDXs, fresh tissue was kept in Roswell Park Memorial Institute (RPMI) 1640 medium on ice for transport and 27 mm^3^ chunks were transplanted in a sterile fashion subcutaneously in the flank of female NOD SCID mice (Envigo, Prattville, AL) under anesthesia with 3% inhalational isoflurane as previously described^26,27^. Mice were maintained in specific pathogen free housing. Tumor volumes were measured with calipers and calculated with the standard formula (width^2^ × length)/2, where the length is the largest measurement. When tumors reached 2000 mm^3^, they were harvested, chopped, and sequentially implanted from mouse to mouse to expand xenograft numbers. Separate portions of the tumor were dissociated to be utilized for experiments. COA3 was a primary tumor from a female child and acquired before treatment. COA6 was a primary tumor from a male child and acquired before treatment. Both were *MYCN* amplified tumors and classified as high-risk disease.

### Antibodies and reagents

Antibodies utilized included rabbit monoclonal anti-Sox2 (2748S) and anti-Nanog (3580S) from Cell Signaling (Danvers, MA), anti-phospho-FAK (Tyr397, Y397) from Invitrogen (Waltham, MA), anti-FAK(C-20) (sc-558) from Santa Cruz (Santa Cruz Biotechnology, Dallas, TX), anti-OCT4 (ab18976) from Abcam (Cambridge, MA), anti-p53 (clone BP53-12, 05-224) from Millipore (Millipore Sigma, St. Louis, MO), and mouse monoclonal β-actin (A1978) from Sigma Aldrich (Millipore Sigma, St. Louis, MO). PF-573,228 (PF) was obtained from Santa Cruz, and 1,2,4,5-benzene tetraamine tetrahydrochloride (Y15) was obtained from Sigma.

### Immunoblotting

Radioimmunoprecipitation (RIPA) buffer supplemented with phenylmethane-sulfonylfluoride (PMSF) (Sigma), protease inhibitor (Sigma), and phosphatase inhibitor (Sigma) was used to make whole cell lysates as previously described^28^. Protein concentrations were determined using a Micro BCA Protein Assay Kit (Thermo Fisher Scientific, Waltham, MA), and samples were loaded into sodium dodecyl sulfate polyacrylamide (SDS-PAGE) gels adjacent to the Precision Plus Protein Kaleidoscope molecular weight marker (Bio-Rad, Hercules, CA). Proteins were separated via electrophoresis and gels transferred to blotting membrane using Trans-Blot Turbo Transfer System (Bio-Rad) per manufacturer’s protocol. Antibodies were utilized per manufacturers’ instructions. Immunoblots were developed with Immobilon Classico or Crescendo Western HRP Substrate (EMD Millipore, Millipore Sigma, Burlington, MA). Blots were stripped with stripping solution (Bio-Rad) at 65 °C for 20 minutes and then re-probed with selected antibodies. Equal protein loading was confirmed using β-actin.

### Immunohistochemistry

As previously described^26^ specimens were sectioned into 6 µm sections and baked at 70 °C for one hour on positive slides. Slides were deparaffinized, steamed, quenched with 3% hydrogen peroxide, and blocked with blocking buffer [bovine serum albumin (BSA), powdered milk, Triton X-100, phosphate buffered saline (PBS)] for 30 minutes at 4 °C. The primary antibody, neuron specific enolase (rabbit polyclonal, 1:300, ab71321, Abcam), was added and incubated overnight in a humidity chamber at 4 °C. After washing with PBS, the secondary antibody for rabbit (R.T.U. biotinylated universal antibody, Vector Laboratories, Burlingame, CA) was added for 30 minutes at 22 °C. The staining reaction was developed for 30 minutes at room temperature with VECTASTAIN Elite ABC reagent (PK-7100, Vector Laboratories) and Metal Enhanced DAB Substrate (Thermo Fisher Scientific) for 2 minutes. Slides were counterstained with hematoxylin. A negative control (rabbit IgG, 1 µg/mL, EMD Millipore) was included with each run.

### Measurement of mRNA expression using real-time PCR (qPCR)

Total cellular RNA was extracted using the RNeasy kit (Qiagen) according to the manufacturer’s protocol. For synthesis of cDNA, 5 μg of RNA was used in a 20-μl reaction mixture utilizing an iScript cDNA Synthesis kit (Bio-Rad) according to the supplier’s instructions. Resulting reverse transcription products were stored at −20 °C until further use. For quantitative real-time PCR, SsoAdvanced™ SYBR® Green Supermix (Bio-Rad) was utilized according to manufacturer’s protocol. Probes specific for the transcription factors Oct4, Nanog, and Sox2 as well as for actin were obtained (Applied Biosystems, Thermo Fisher Scientific). qPCR was performed with 10 ng cDNA in 50 μL reaction volume. Amplification was done using an Applied Biosystems 7900HT cycler (Applied Biosystems). Cycling conditions were 95 °C for 30 sec, 95 °C for 5 sec followed by 40-cycle amplification at 95 °C for 15 s and 65 °C for 1 min. Experiments were repeated at least three times, and samples were analyzed in triplicate with Actin B utilized as an internal control. Data were calculated utilizing the ΔΔCt method^29^ and are reported as mean fold change ± standard error of the mean (SEM).

### Cell survival and proliferation

Following tumor dissociation, cells were suspended in NB media and counted. To assess cell survival, alamarBlue® colorimetric assay (Thermo Fisher Scientific) was utilized. Cells (1.5 × 10^4^) were plated on 96-well plates and treated the following day with PF or Y15 in increasing concentrations, or an equivalent concentration of vehicle (DMSO). After 24 hours, 10 μL of alamarBlue® dye (Thermo Fisher Scientific) was added and absorbance was read at 570 nm and 600 nm on a microplate reader (Epoch Microplate Spectrophotometer, BioTek Instruments, Winooski, VT). Proliferation was evaluated using the colorimetric CellTiter96® Aqueous Non-Radioactive Cell Proliferation Assay (Promega, Madison, WI). Cells (5 × 10^3^) were plated and treated the following day with vehicle, PF, or Y15 in increasing concentrations. CellTiter96® dye (10 μL, Promega) was added 24 hours later and absorbance read at 490 nm. Results in survival and proliferation from at least three biologic replicates were reported as fold change ± SEM.

### Cell cycle

In 6-well plates, 3 × 10^6^ cells were plated and treated with increasing doses of PF (0, 5, 20 μM) or Y15 (0, 5, 10 μM) for three days. Accutase was used to create a single cell suspension, and then those cells were washed with PBS and fixed with ethanol. Following a second wash with PBS, cells were stained with propidium iodide (Invitrogen), 0.1% TritonX (Active Motif, Carlsbad, CA), and RNAse A (0.1 mg/mL, Qiagen, Valencia, CA), and cell cycle was analyzed via FACSCalibur^TM^ Flow Cytometer (BD Biosciences). Data were analyzed using ModFit LT software (Verity Software House Inc., Topsham, ME). Data presented are the results of at least three biologic replicates.

### Migration and invasion

For migration and invasion assays, 24-well 8 μm pore Corning® Transwell® plates (Millipore Sigma) were utilized. For COA3 in both assays, the bottoms of the inserts were coated with collagen I (10 µg/mL, MP Biomedicals, Santa Ana, CA) overnight at 37 °C and then washed with PBS. For the invasion assays, the inside of the 8 µm micropore Transwell® inserts were coated with Matrigel™ (1 mg/mL, 50 µL; BD Biosciences, Franklin Lakes, NJ) overnight at 37 °C and then washed with PBS. COA3 cells (3 × 10^6^) pretreated for 24 hours with vehicle, PF or Y15 were added to each insert and allowed to migrate for three days or invade for one week. For migration and invasion of COA6 cells, the bottoms of the inserts were coated with laminin (Trevigen, Gaithersburg, MD), incubated overnight at 37 °C, and then washed with PBS prior to plating. COA6 cells (4 × 10^4^) were pretreated for 24 hours with vehicle, PF, or Y15 and added to each insert. Cells were allowed to migrate for 72 hours. For invasion, the top of the insert was coated with Matrigel™ (1 mg/mL, 50 µL; BD Biosciences) overnight at 37 °C and then washed with PBS. Cells were allowed to invade for a week. Inserts were then fixed with 4% paraformaldehyde and stained with crystal violet and analyzed using Image J (https://imagej.nih.gov/ij). Results from at least three biologic replicates were reported as fold change in number of cells migrating or invading ± SEM.

### *In vitro* limiting dilution sphere assay

To determine if FAK supported the SCLCC phenotype, sphere forming ability was measured using *in vitro* limiting dilution assays. Conditioned NB media was harvested after 24–48 hours of culture with untreated cells. Single cells were plated in conditioned NB media onto 96-well low attachment plates using serial dilutions with 5000, 1000, 100, 50, 20, 10, and 1 cell per well. Cells were then treated with PF (0, 5 μM) or Y15 (0, 5 μM). One week later the number of wells containing tumor spheres in each row was counted by a single researcher and the results were analyzed using the extreme limiting dilution analysis software (http://bioinf.wehi.edu.au/software/elda/).

### Magnetic cell sorting

Separation of the CD133-enriched from the CD133-depleted cell population was performed using the Miltenyi Biotec CD133 MicroBead Kit (Miltenyi Biotech). After cells were harvested from xenograft tumors, they were washed and suspended in MACSBuffer Rinsing solution (Miltenyi). Cells were blocked for five minutes with FcR Blocking reagent (Miltenyi) and then stained for fifteen minutes with CD133 Microbeads (Miltneyi). Following staining, cells were washed with additional buffer twice and then passed through MS Columns (Miltneyi), using one column for every 20–30 million cells. Negative cells were collected from flow-through and positive cells were subsequently plunged from the magnetic column. Cells were then suspended in NB media for experimentation. Separation efficiency was determined using a check reagent and FACS analysis.

### Data analysis

All experiments were repeated with at least three biologic replicates and data were reported as mean ± standard error of the mean (SEM). Student’s t-test or ANOVA was used as appropriate, with p ≤ 0.05 determined to be statistically significant.

## Supplementary information

Supplementary Data

Supplementary Data

## References

[1] Smith, V. & Foster, J. High-Risk Neuroblastoma Treatment Review. *Children (Basel)*, **5**(9). Epub 2018/08/28. 10.3390/children5090114, PubMed PMID: 30154341; PMCID: PMC6162495 (2018). 10.3390/children5090114 PMC6162495 30154341

[2] Owens, L. V. *et al*. Overexpression of the focal adhesion kinase (p125FAK) in invasive human tumors. *Cancer Res*. **55**(13), 2752–5, PubMed PMID: 7796399 (1995). 7796399

[3] Waters, A. M. & Beierle, E. A. The interaction between FAK, MYCN, p53 and Mdm2 in neuroblastoma. *Anticancer Agents Med Chem*.**14**(1), 46–51 PubMed PMID: 24041229 (2014). 10.2174/18715206113136660331 24041229

[4] Cooper, J. & Giancotti, F. G. Integrin Signaling in Cancer: Mechanotransduction, Stemness, Epithelial Plasticity, and Therapeutic Resistance. *Cancer Cell*. **35**(3), 347–67, 10.1016/j.ccell.2019.01.007, PubMed PMID: 30889378 (2019). 10.1016/j.ccell.2019.01.007 PMC6684107 30889378

[5] Mitra, S. K., Hanson, D. A. & Schlaepfer, D. D. Focal adhesion kinase: in command and control of cell motility. *Nat Rev Mol Cell Biol*. **6**(1), 56–68, 10.1038/nrm1549. PubMed PMID: 15688067 (2005). 10.1038/nrm1549 15688067

[6] Beierle, E. A. *et al*. Focal adhesion kinase expression in human neuroblastoma: immunohistochemical and real-time PCR analyses. *Clin Cancer Res*. **14**(11), 3299–305, 10.1158/1078-0432.CCR-07-1511, PubMed PMID: 18519756 (2008). 10.1158/1078-0432.CCR-07-1511 18519756

[7] Beierle, E. A. *et al*. Inhibition of focal adhesion kinase decreases tumor growth in human neuroblastoma. *Cell Cycle*.**9**(5), 1005–15, Epub 2010/03/14, 10.4161/cc.9.5.10936, PubMed PMID: 20160475; PMCID: PMC2855768 (2010). 10.4161/cc.9.5.10936 PMC2855768 20160475

[8] Miller, A. L. *et al*. Whole exome sequencing identified sixty-five coding mutations in four neuroblastoma tumors. *Sci Rep*. **7**(1), 17787, 10.1038/s41598-017-17162-y, PubMed PMID: 29259192; PMCID: PMC5736554 (2017). 10.1038/s41598-017-17162-y PMC5736554 29259192

[9] Jung, J., Seol, H. S. & Chang, S. The Generation and Application of Patient-Derived Xenograft Model for Cancer Research. *Cancer Res Treat*. **50**(1), 1–10, Epub 2017/09/13, 10.4143/crt.2017.307, PubMed PMID: 28903551; PMCID: PMC5784646 (2018). 10.4143/crt.2017.307 PMC5784646 28903551

[10] Zhao, J. H., Reiske, H. & Guan, J. L. Regulation of the cell cycle by focal adhesion kinase. *J Cell Biol*.**143**(7), 1997–2008, PubMed PMID: 9864370; PMCID: PMC2175220 (1998). 10.1083/jcb.143.7.1997 PMC2175220 9864370

[11] Megison, M. L., Stewart, J. E., Nabers, H. C., Gillory, L. A. & Beierle, E. A. FAK inhibition decreases cell invasion, migration and metastasis in MYCN amplified neuroblastoma. *Clin Exp Metastasis*. **30**(5), 555–68, Epub 2012/12/04, 10.1007/s10585-012-9560-7, PubMed PMID: 23208732; PMCID: PMC3625446 (2013). 10.1007/s10585-012-9560-7 PMC3625446 23208732

[12] Chambers, I. & Tomlinson, S. R. The transcriptional foundation of pluripotency. *Development*. **136**(14), 2311–22, 10.1242/dev.024398, PubMed PMID: 19542351; PMCID: PMC2729344 (2009). 10.1242/dev.024398 PMC2729344 19542351

[13] Garner, E. F. & Beierle, E. A. Cancer Stem Cells and Their Interaction with the Tumor Microenvironment in Neuroblastoma. *Cancers (Basel)*. **8**(1) Epub 2015/12/31, 10.3390/cancers8010005, PubMed PMID: 26729169; PMCID: PMC4728452 (2015). 10.3390/cancers8010005 PMC4728452 26729169

[14] Braekeveldt, N. & Bexell, D. Patient-derived xenografts as preclinical neuroblastoma models. *Cell Tissue Res*. **372**(2), 233–43, Epub 2017/09/19, 10.1007/s00441-017-2687-8, PubMed PMID: 28924803; PMCID: PMC5915499 (2018). 10.1007/s00441-017-2687-8 PMC5915499 28924803

[15] Williams, A. P. *et al*. Corruption of neuroblastoma patient derived xenografts with human T cell lymphoma. *J Pediatr Surg*. Epub 2018/10/22, 10.1016/j.jpedsurg.2018.10.051, PubMed PMID: 30391152 (2018). 10.1016/j.jpedsurg.2018.10.051 PMC6476711 30391152

[16] Golubovskaya, V. M. *et al*. A small molecule inhibitor, 1,2,4,5-benzenetetraamine tetrahydrochloride, targeting the y397 site of focal adhesion kinase decreases tumor growth. *J Med Chem*. **51**(23), 7405–16, 10.1021/jm800483v, PubMed PMID: 18989950; PMCID: PMC2662449 (2008). 10.1021/jm800483v PMC2662449 18989950

[17] Hochwald, S. N. *et al*. A novel small molecule inhibitor of FAK decreases growth of human pancreatic cancer. *Cell Cycle*. **8**(15), 2435–43, Epub 2009/08/01, 10.4161/cc.8.15.9145, PubMed PMID: 19571674; PMCID: PMC4824314 (2009). 10.4161/cc.8.15.9145 PMC4824314 19571674

[18] Weiss, W. A., Taylor, S. S. & Shokat, K. M. Recognizing and exploiting differences between RNAi and small-molecule inhibitors. *Nat Chem Biol*.**3**(12), 739–44, 10.1038/nchembio1207-739, PubMed PMID: 18007642; PMCID: PMC2924165 (2007). 10.1038/nchembio1207-739 PMC2924165 18007642

[19] Alisi, A., Cho, W. C., Locatelli, F. & Fruci, D. Multidrug resistance and cancer stem cells in neuroblastoma and hepatoblastoma. *Int J Mol Sci*. **14**(12), 24706–25, Epub 2013/12/18, 10.3390/ijms141224706, PubMed PMID: 24351843; PMCID: PMC3876137 (2013). 10.3390/ijms141224706 PMC3876137 24351843

[20] Chen, K., Huang, Y. H. & Chen, J. L. Understanding and targeting cancer stem cells: therapeutic implications and challenges. *Acta Pharmacol Sin*. **34**(6), 732–40, Epub 2013/05/20, 10.1038/aps.2013.27, PubMed PMID: 23685952; PMCID: PMC3674516 (2013). 10.1038/aps.2013.27 PMC3674516 23685952

[21] Schober, M. & Fuchs, E. Tumor-initiating stem cells of squamous cell carcinomas and their control by TGF-β and integrin/focal adhesion kinase (FAK) signaling. *Proc Natl Acad Sci USA* **108**(26), 10544–9, Epub 2011/06/13, 10.1073/pnas.1107807108, PubMed PMID: 21670270; PMCID: PMC3127891 (2011). 10.1073/pnas.1107807108 PMC3127891 21670270

[22] Luo, M. *et al*. Distinct FAK activities determine progenitor and mammary stem cell characteristics. *Cancer Res*. **73**(17), 5591–602, Epub 2013/07/05, 10.1158/0008-5472.CAN-13-1351, PubMed PMID: 23832665; PMCID: PMC3766468 (2013). 10.1158/0008-5472.CAN-13-1351 PMC3766468 23832665

[23] Oh, L., Hafsi, H., Hainaut, P. & Ariffin, H. p53, stem cell biology and childhood blastomas. *Curr Opin Oncol*. **31**(2), 84–91, 10.1097/CCO.0000000000000504, PubMed PMID: 30585860 (2019). 10.1097/CCO.0000000000000504 30585860

[24] Jain, A. K. & Barton, M. C. p53: emerging roles in stem cells, development and beyond. *Development*. **145**(8), Epub 2018/04/13, 10.1242/dev.158360, PubMed PMID: 29654218 (2018). 10.1242/dev.158360 29654218

[25] Gillory, L. A., Stewart, J. E., Megison, M. L., Waters, A. M. & Beierle, E. A. FAK and p53 Synergistically Decrease Neuroblastoma Cell Survival. *The Journal of surgical research*. **196**(2), 339–49, 10.1016/j.jss.2015.03.021, PubMed PMID: PMC4442704 (2015). 10.1016/j.jss.2015.03.021 PMC4442704 25862488

[26] Stafman, L. L. *et al*. Targeting PIM kinase as a therapeutic strategy in human hepatoblastoma. *Oncotarget*. **9**(32), 22665–79, 10.18632/oncotarget.25205 PubMed PMID: 29854306; PMCID: PMC5978256 (2018). 10.18632/oncotarget.25205 PMC5978256 29854306

[27] Stafman, L. L. *et al*. The presence of PIM3 increases hepatoblastoma tumorigenesis and tumor initiating cell phenotype and is associated with decreased patient survival. *J Pediatr Surg*. **54**(6), 1206–13, 10.1016/j.jpedsurg.2019.02.029, PubMed PMID: 30898394; PMCID: PMC6545248 (2019). 10.1016/j.jpedsurg.2019.02.029 PMC6545248 30898394

[28] Beierle, E. A. *et al*. N-MYC regulates focal adhesion kinase expression in human neuroblastoma. *J Biol Chem*. **282**(17), 12503–16, Epub 2007/02/27, 10.1074/jbc.M701450200, PubMed PMID: 17327229 (2007). 10.1074/jbc.M701450200 17327229

[29] Winer, J., Jung, C. K., Shackel, I. & Williams, P. M. Development and validation of real-time quantitative reverse transcriptase-polymerase chain reaction for monitoring gene expression in cardiac myocytes *in vitro*. *Anal Biochem*. **270**(1), 41–9, 10.1006/abio.1999.4085, PubMed PMID: 10328763 (1999). 10.1006/abio.1999.4085 10328763

