# Supplementary material for: Focal Adhesion Kinase Inhibition Contributes to Tumor Cell Survival and Motility in Neuroblastoma Patient-Derived Xenografts
Source: Sci Rep. 2019 Sep 13;9:13259. doi: 10.1038/s41598-019-49853-z (PMC6744403; doi:10.1038/s41598-019-49853-z)
Supplement: Supplementary file 1 — Supplementary Data [file 41598_2019_49853_MOESM1_ESM.pdf]

Focal Adhesion Kinase Inhibition Contributes to Tumor Cell Survival and Motility in Neuroblastoma Patient-Derived Xenografts

Laura L. Stafman[1*], Adele P. Williams[1*], Raoud Marayati[1], Jamie M. Aye[2], Hooper R. Markert[1], Evan F. Garner[1], Colin H. Quinn[1], Shoeb B. Lallani[3], Jerry E. Stewart[1], Karina J. Yoon[4], Kimberly Whelan[2], Elizabeth A. Beierle[1]

Supplementary Data Fig. 1

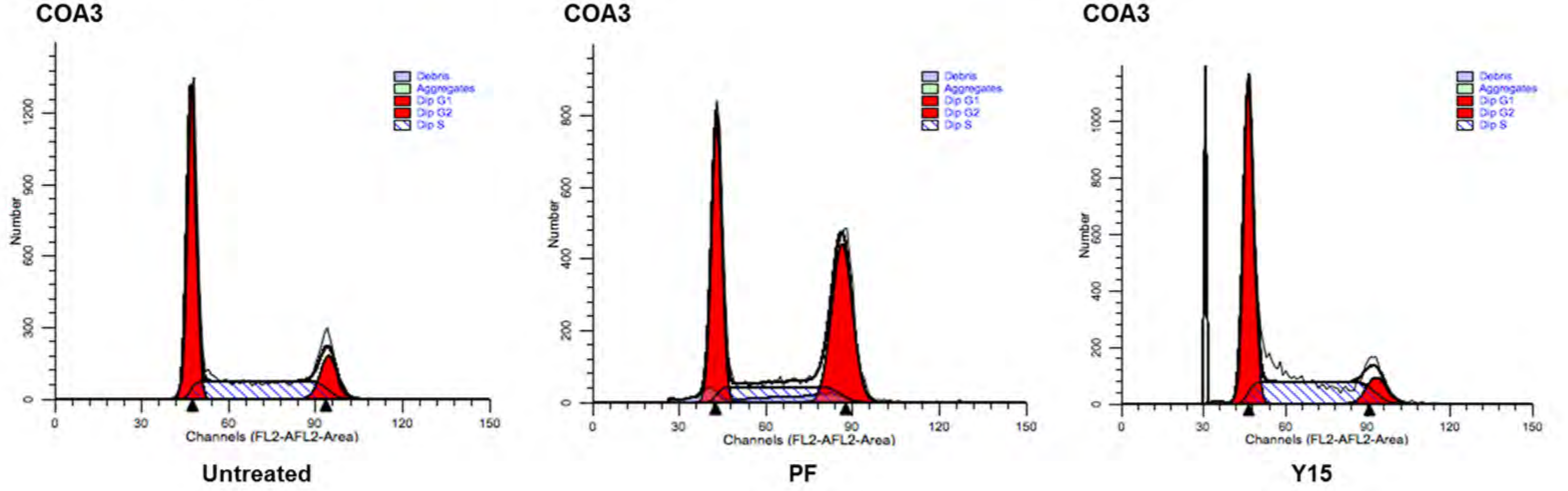

Supplementary Data Fig. 2

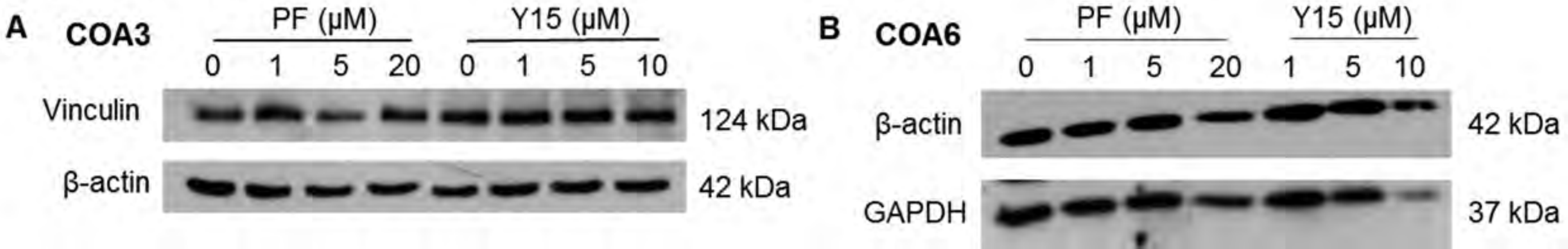

Supplementary Data

Figure 1: FAK inhibition diminished progression through the cell cycle. COA3 cells (3×10^6) were treated with vehicle, PF (20 μM) or Y15 (10 μM) for 72 hours. Cells were stained with propidium iodide and cell cycle was analyzed via flow cytometry. Representative histograms are presented. FAK inhibition led to an increase in number of cells in the G1 phase and a decrease of those in S phase that was statistically significant, representing failure to progress through the cell cycle.

Figure 2: FAK inhibition does not affect protein levels of β-actin. Immunoblotting of tumor lysates for Vinculin, GAPDH, and β-actin in both COA3 and COA6 cells following treatment with FAK inhibitors PF and Y15 demonstrated equal protein loading.
