# Supplementary material for: Focal Adhesion Kinase Inhibition Contributes to Tumor Cell Survival and Motility in Neuroblastoma Patient-Derived Xenografts
Source: Sci Rep. 2019 Sep 13;9:13259. doi: 10.1038/s41598-019-49853-z (PMC6744403; doi:10.1038/s41598-019-49853-z)
Supplement: Supplementary file 2 — Supplementary Data [file 41598_2019_49853_MOESM2_ESM.pdf]

Focal Adhesion Kinase Inhibition Contributes to Tumor Cell Survival and Motility in Neuroblastoma Patient-Derived Xenografts

Laura L. Stafman[1*], Adele P. Williams[1*], Raoud Marayati[1], Jamie M. Aye[2], Hooper R. Markert[1], Evan F. Garner[1], Colin H. Quinn[1], Shoeb B. Lallani[3], Jerry E. Stewart[1], Karina J. Yoon[4], Kimberly Whelan[2], Elizabeth A. Beierle[1]

Supplementary
Uncut Western Blots

Fig 1B

COA3 pFAK

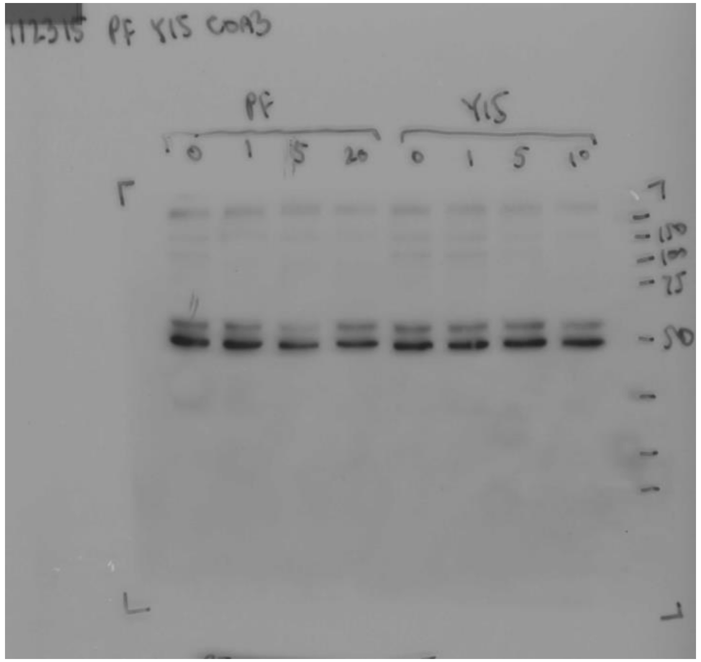

FAK

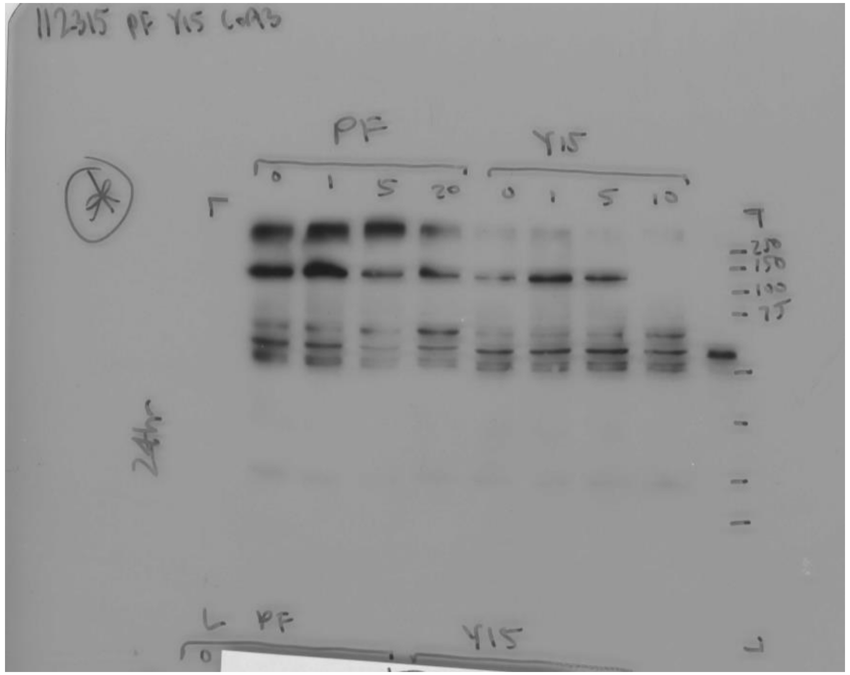

Actin

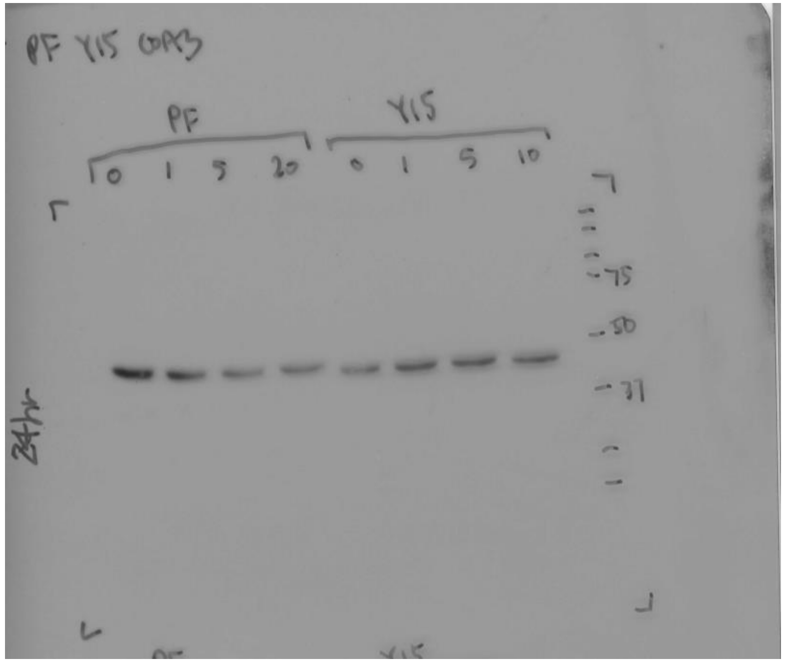

COA6 pFAK

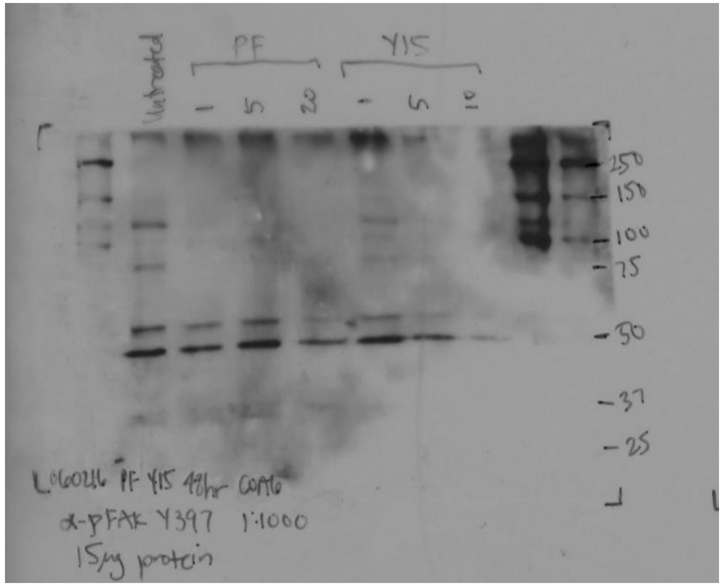

FAK

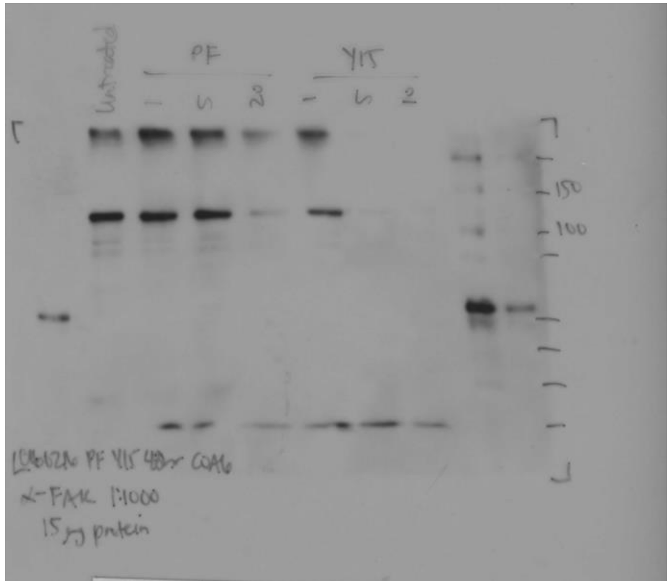

Actin

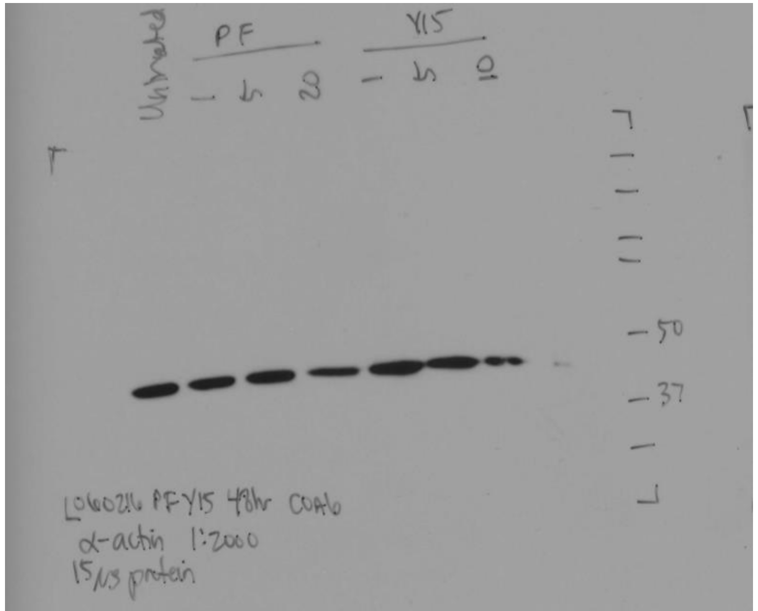

Fig.5A

Coa3

Nanog Sox2 Oct4

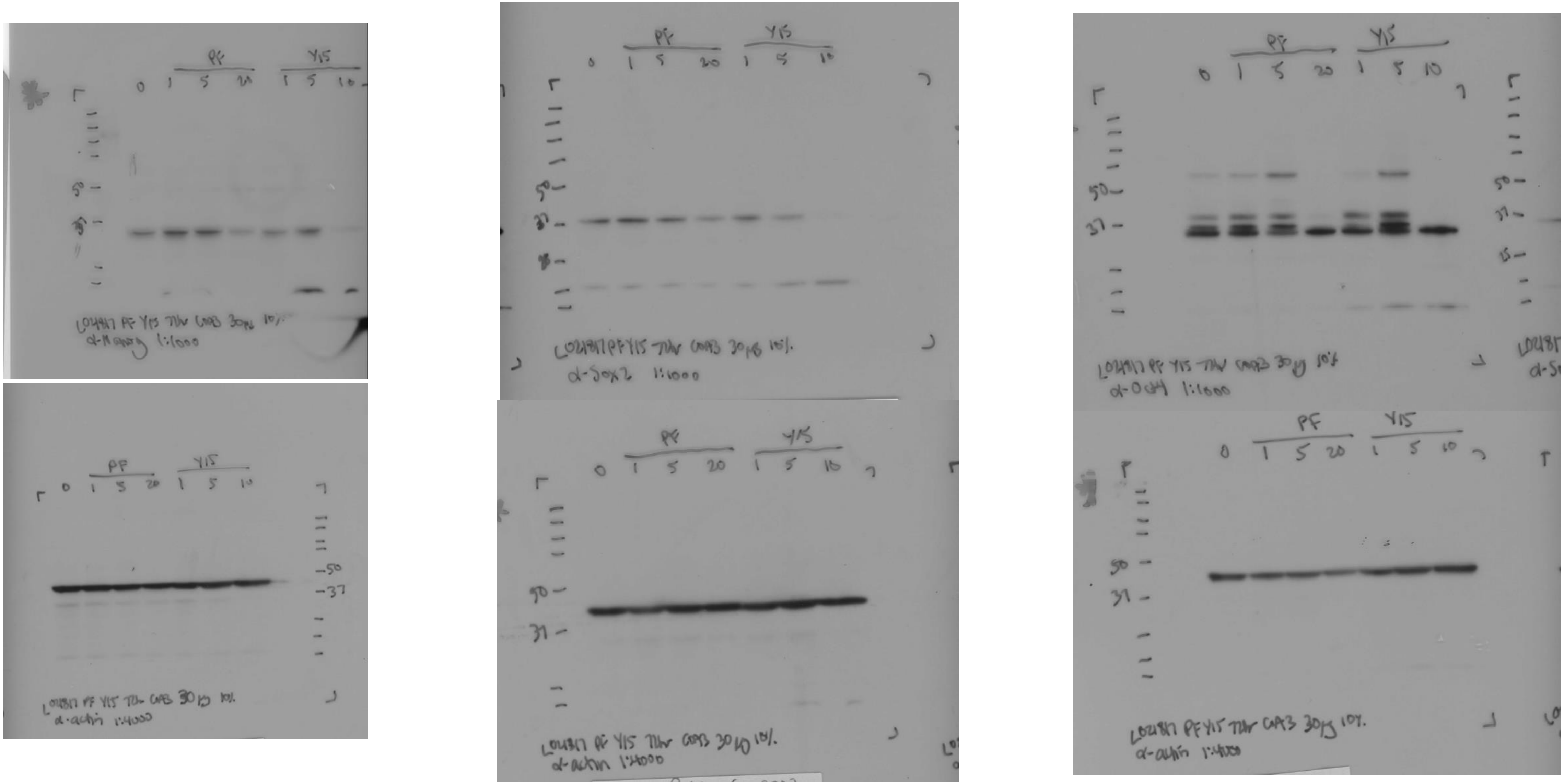

Fig. 5A

Coa3

Nanog Sox2 Oct4

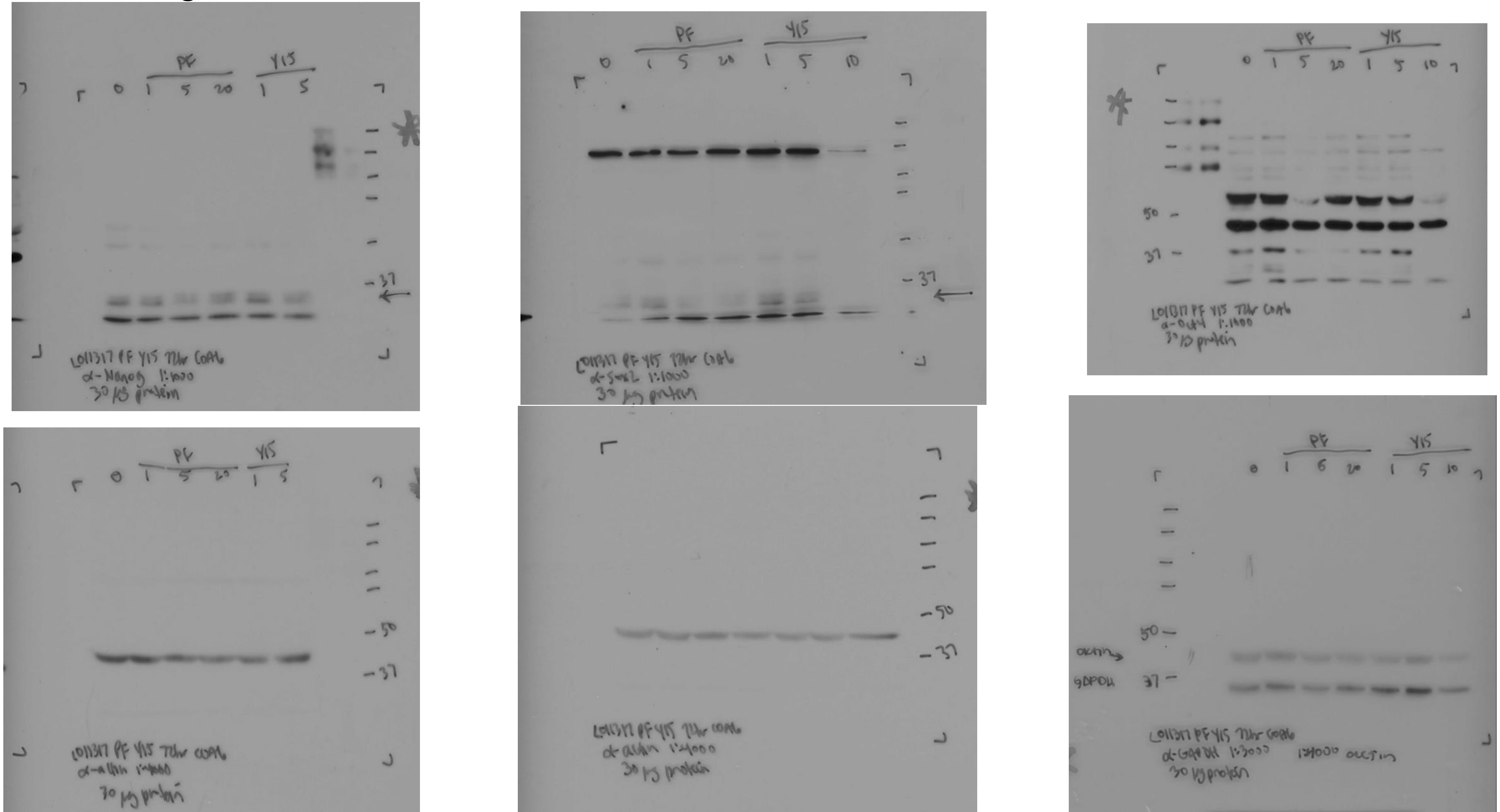

Supplemental Data Fig 3

COA6 P53

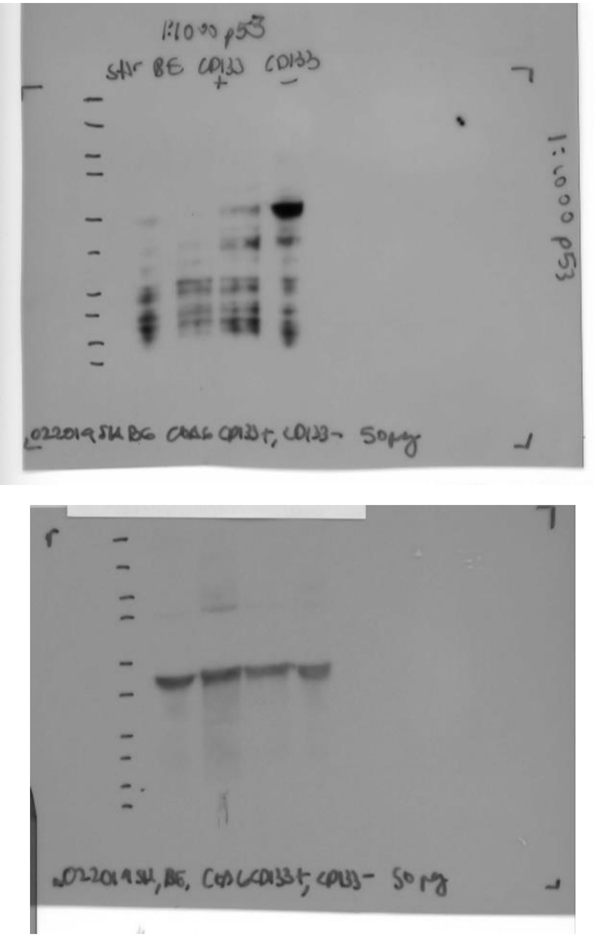

COA6 pFAK, FAK, p53, actin

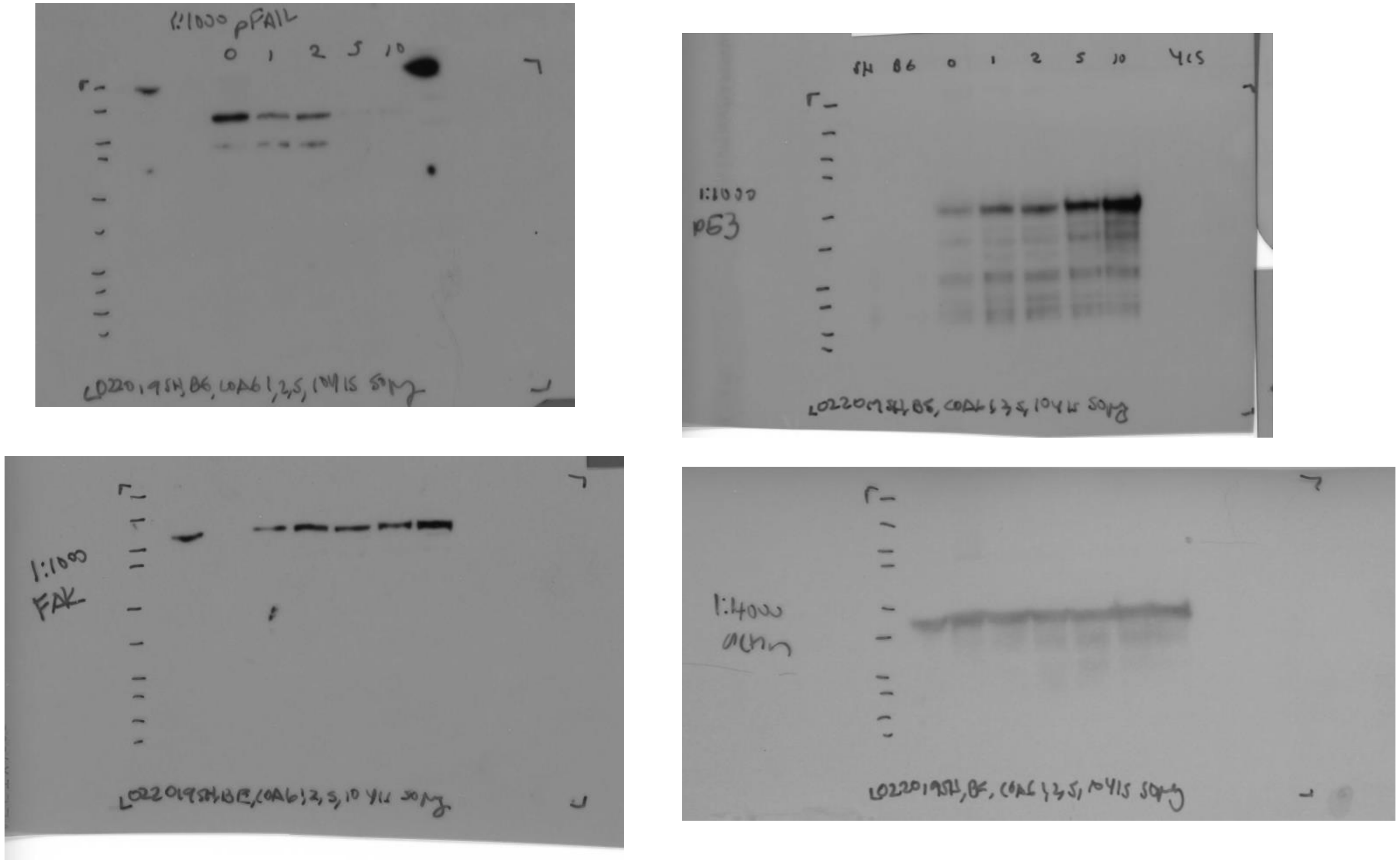

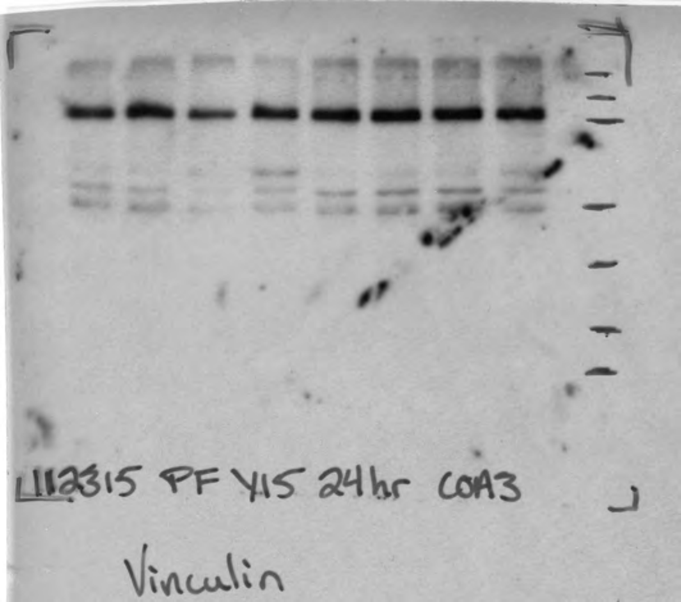
112315 PF Y15 24hr COA3
Vinculin

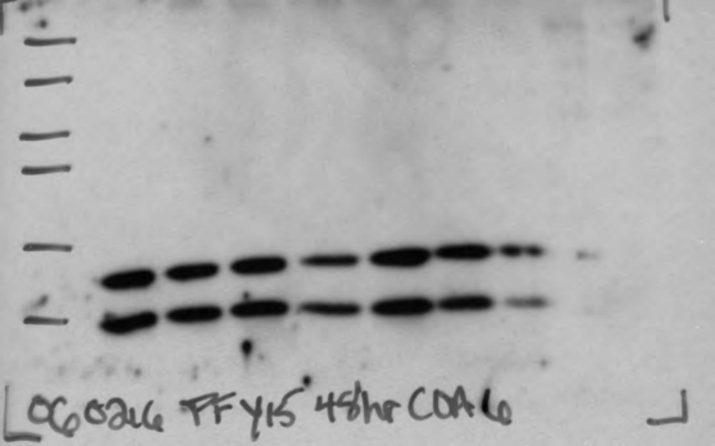
060216 FFY15 48hr COA6
